# Hydroxyapatite-poly(d,l-lactide) Nanografts. Synthesis and Characterization as Bone Cement Additives

**DOI:** 10.3390/molecules26020424

**Published:** 2021-01-15

**Authors:** Kristina L. Goranova, Anne Kathrine Kattenhøj Sloth Overgaard, Ivan Gitsov

**Affiliations:** 1 Department of Chemistry, State University of New York-ESF, Syracuse, NY 13210, USA; klgorano@syr.edu (K.L.G.); akovergaard@yahoo.dk (A.K.K.S.O.); 2 Coloplast, Holtedam 1-3, DK-3050 Humlebǽk, Denmark; 3 The Michael M. Szwarc Polymer Research Institute, Syracuse, NY 13210, USA

**Keywords:** hydroxyapatite, lactide, ring-opening polymerization, polyesters, biomaterials, bone cement

## Abstract

This paper reports the creation of hydroxyapatite/polyester nanografts by “graft-from” polymerization of d,l-lactide with [Ca_5_(OH)(PO_4_)_3_]_2_ as the initiator and tin(II)-2-ethylhexanoate as the catalyst. Model polymerizations were performed with cyclooctanol as initiator to confirm the grafting on the surface of the hydroxyapatite nanocrystals. Polymers with the highest molecular mass (M_n_) between 4250 Da (cyclooctanol) and 6100 Da (hydroxyapatite) were produced. In both cases the molecular mass distributions of the polymers formed were monomodal. The materials obtained were characterized by size-exclusion chromatography, NMR and FT-IR spectroscopy, and thermal methods. Their suitability as additives for commercial bone cement (Simplex P Speedset, Stryker Orthopaedics) has been confirmed by thermal analysis techniques and mechanical testing. The results obtained show that addition of the hydroxyapatite/ polyester nanografts improved both thermal and mechanical properties of the bone cement.

## 1. Introduction

Acrylic bone cement has been the standard biomaterial for prosthesis fixation during total hip arthroplasty for more than 50 years [1]. The main function of the cement is to stabilize the metal prosthesis by filling the gap between the prosthesis and the surrounding bone, and to transmit the load from the prosthesis to the bone. One of the major drawbacks of the acrylic material, however, is its failure to induce a direct bond to the adjacent bone. The formation of a fibrous layer between the bone surface and the cement is one of the main reasons for implant failure [2]. The increasing number of hip replacement operations (approximately 400,000 annually in the USA with that number expected to rise to 572,000 by 2030) [3,4] and the strong impact on the quality of life of the affected individuals drive the need to develop new materials with significantly improved properties.

Some of the efforts at improving the properties of acrylic bone cements currently in use involve addition of bioactive fillers such as hydroxyapatite (HA) to the standard formulation. HA constitutes the crystalline element of bone [5] and has been in use as a filler and component of composite materials for bone replacement [6,7]. The simple mixing of inorganic and organic materials, however, causes the formation of particle aggregates in the resulting mixtures and increases susceptibility to failure [6]. Improvement of the interface contact between HA and polymer matrix has been attempted through surface modification of HA with dodecyl alcohol [6], silane coupling agents [7,8,9], isocyanate [10,11,12,13] and poly(acids) [14,15]. The “grafting-from” approach for chemical modification of the surface of HA is an alternative strategy, which employs polymerization initiated by the reactive groups at the surface of HA particles. The technique has been used by Chen et al. in an attempt to modify HA with poly(l-lactide) [16,17,18,19]. Due to their biocompatibility and biodegradability [20,21], poly(lactides) have been used in a great variety of biomedical applications including HA compositions [22,23,24]. These polyesters can be conveniently synthesized by ring-opening polymerization of lactides in the presence of tin(II)-2-ethylhexanoate (SnOct_2_) [25,26]. Besides high efficiency [27], this catalyst also has low toxicity being a Federal Drug Agency approved additive [28], which is of essential importance for the synthesis of materials for biomedical applications.

Despite the fact that the polymerization of lactides from various HA materials has been described in previous publications [16,17,18,19,29,30], a systematic investigation of the process has not been performed. Thus, the main goal of this study is the detailed exploration of the influence of the polymerization conditions (type of HA, monomer/initiator ratio) on the density of grafting, the length of the grafted fragments and the physical properties of the graft polymers formed. Model polymerizations with cyclooctanol were performed under the same reaction conditions to confirm the grafting on the HA surface and to reveal the peculiarities of the heterogeneous HA polymerization process. Finally the compatibility of the HA/poly(d,l-lactide) hybrids with commercial bone cement formulations was studied in order to evaluate their application potential.

## 2. Results and Discussion

**Scheme 1:**
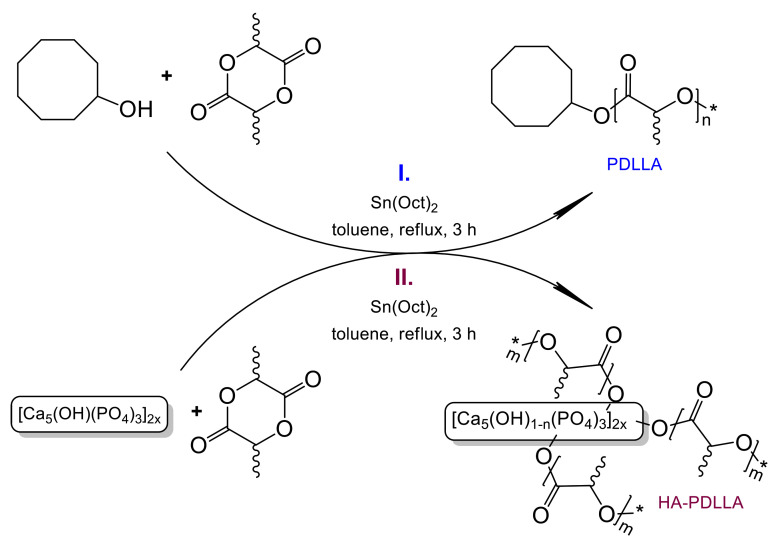
Ring-opening polymerizations of d,l-lactide initiated by Sn(Oct)_2_/cyclooctanol (I) and Sn(Oct)_2_/hydroxyapatite (II).

A series of polymerization experiments was conducted in order to obtain a controlled ring-opening polymerization of d,l-lactide. In a model reaction, this cyclic ester has been polymerized with cyclooctanol as the initiator and SnOct_2_ as the catalyst according to the reaction pathway depicted in Scheme 1(I). Polymerizations were carried out according to the procedure described in the Experimental Part at monomer-to catalyst ratio ((M)/(C)) between 10/1 and 200/1. All reactions were performed in triplicate to explore and verify the ability of the synthetic method to yield materials with reproducible molecular mass characteristics.

A series of polymerizations under exactly the same conditions was conducted using hydroxyapatite, Ca_10_(PO_4_)_6_(OH)_2,_ as a source of initiating hydroxyl groups, Scheme 1(II). 

**Figure 1:**
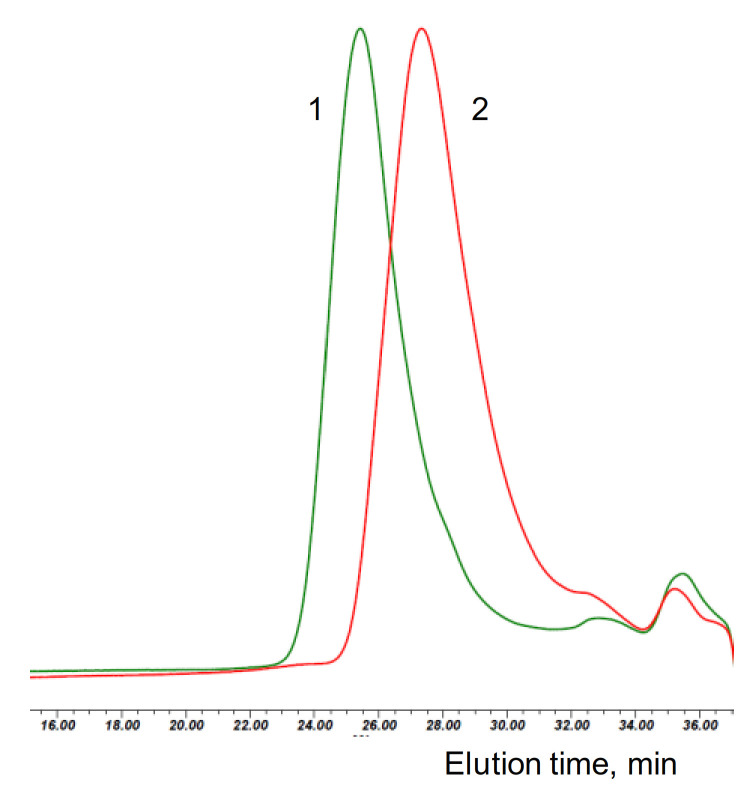
SEC eluograms of the polymerization mixtures obtained with d,l-lactide and hydroxyapatite (HA)/Sn(Oct)_2_ (green trace, 1) and Cyclooctanol/Sn(Oct)_2_ (red trace, 2) with (M)/(C) = 150.

The results obtained show that the cyclooctanol/Sn(Oct)_2_ initiating system affords polymers (assigned as “PDLLA #”, the number indicating the monomer to catalyst ratio) with yields between 93% and 97%, and rather reproducible molecular mass characteristics, the molecular mass, M_n_, ranging from 540 Da to 4250 Da (Table 1 and Figure 1). The polymers are mostly free of oligomer byproducts and their molecular mass increases with the increase in the monomer/catalyst ratio as expected for this type of polymerization system.

During the course of the polymerization initiated by hydroxyapatite (HA), the HA crystals, which were initially insoluble in toluene, went into solution and the reaction appeared to proceed in a homogenous fashion. This could be interpreted as an indication that the propagating poly(d,l-lactide) chains, which are freely soluble in the polymerization medium, are grafted onto the HA and, upon reaching certain “critical” mass, lead to its gradual dissolution. We estimate this mass to be above 1000 Da since all HA-PDDLA samples in Table 1 dissolved during the polymerization. The typical values of M_n_ for this series are also shown in Table 1.

Under identical monomer/catalyst ratios and the same reaction conditions, the ring-opening polymerization of d,l-lactide with HA as initiator consistently produces polymers in almost quantitative yields (95–97%) and with higher molecular mass, compared to the cyclooctanol-initiated process. The polymers are assigned as “HA-PDLLA #”, the number again indicating the monomer to catalyst ratio. Since this ratio is the same the increase in the molecular mass could be attributed to the presence of multiple initiation sites (OH groups) anchored at the same HA particle. The molecular masses are also quite reproducible, the deviations presumably reflecting the initial heterogeneity of the polymerization system. It should be noted that the change of the initial polymerization solvent from triethylene glycol dimethyl ether (triglyme) to toluene led to a significant improvement in polymer yields (Table S1 vs. Table 1).

Figure 1 shows the size exclusion chromatography (SEC) traces of the reaction mixtures of selected cyclooctanol/Sn(Oct)_2_ and HA/Sn(Oct)_2_ polymerizations. Notably the main polymer peaks in the chromatograms from the HA polymerizations are monodisperse—a clear indication for the simultaneous initiation by all accessible OH groups on the surface of HA particles (see also Figure S1 in the Supporting Information).

**Figure 2:**
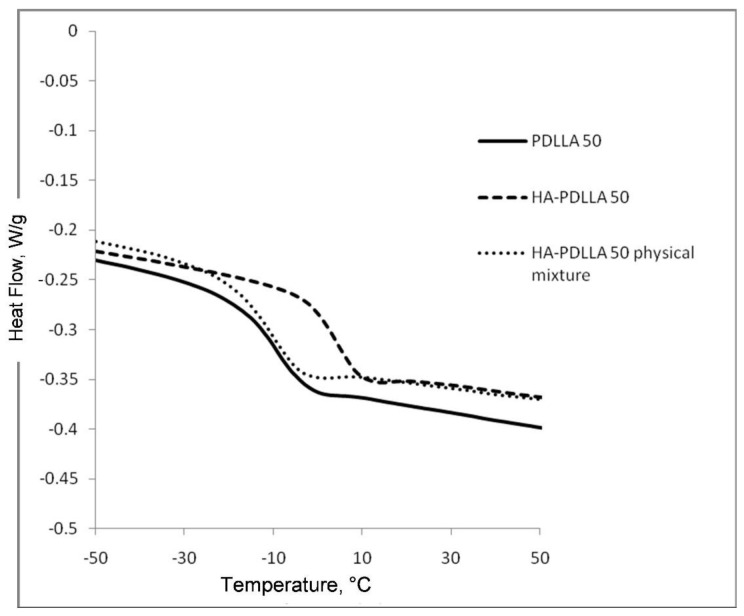
Differential scanning calorimetry (DSC) traces of polyesters obtained with (M)/(C) = 50: PDLLA homopolymer (PDLLA 50), HA-PDLLA graft (HA-PDLLA 50), and HA-PDLLA 50 physical mixture.

**Figure 3:**
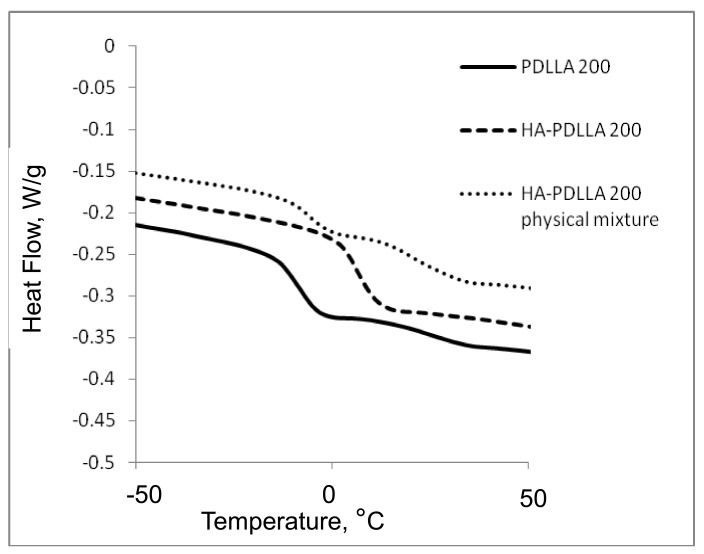
DSC traces of polyesters obtained with (M)/(C) = 200: PDLLA homopolymer (PDLLA 200), HA-PDLLA graft (HA-PDLLA 200), and HA-PDLLA 200 physical mixture.

The attachment of the polymer to the surface of the hydroxyapatite particles was further confirmed by differential scanning calorimetry (DSC), Figure 2 and Figure 3. The thermograms of poly (d,l-lactides) produced with cyclooctanol as initiator showed well defined glass transitions. The observed insignificant difference in the glass transition temperature (T_g_) from −6 °C for PDLLA 50 to −7 °C for PDLLA 200 shows a lack of molecular mass effect on the latter (see PDLLA data in Table 1). However, the observed notable shift in the T_g_ of all HA-PDLLA polymers compared to their PDLLA counterparts (an example is shown in Figure S2) could not be explained by a simple increase in the molecular mass as evidenced by the thermal characteristics of the physical mixtures with compositions identical to those of the HA grafts (HA is physically mixed with the corresponding PDLLA to produce a composition identical to the graft), Figure 2 and Figure 3. The T_g_s of these physical mixtures are practically equal to the T_g_s of the corresponding PDLLA. Therefore the consistently higher T_g_ values for the HA materials are mostly caused by the decreased segmental motion of the poly(d,l-lactide) chains covalently bound to the surface of the HA.

**Figure 4:**
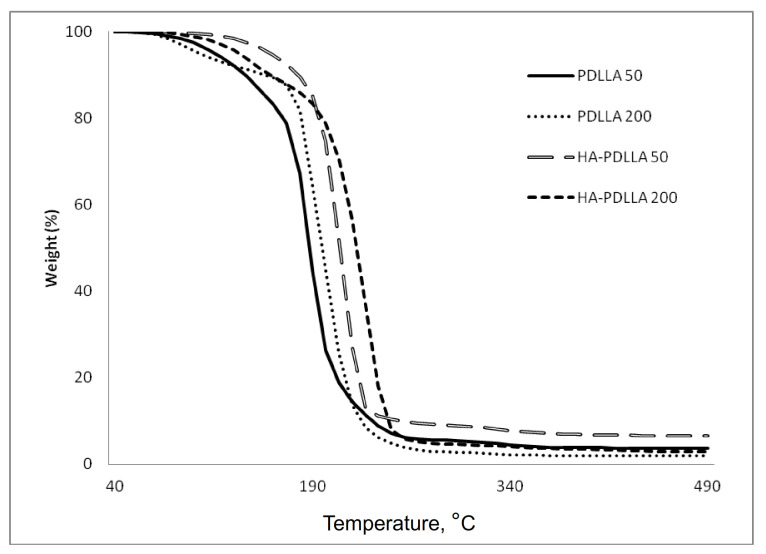
TGA thermograms of PDLLA 50, PDLLA 200 and the corresponding grafts, HA-PDLLA 50 and HA-PDLLA 200.

A similar trend was also observed in the thermal degradation pattern investigated by thermal gravimetric analyses, Figure 4. It is seen that the degradation temperature of the HA polymers is higher by 6 °C to 20 °C compared to the values of the linear PDLLA (from 200 °C to 206 °C for PDLLA 200 and HA-PDLLA 200 and from 189 °C to 209 °C for PDLLA 50 and HA-PDLLA 50, respectively).

**Figure 5:**
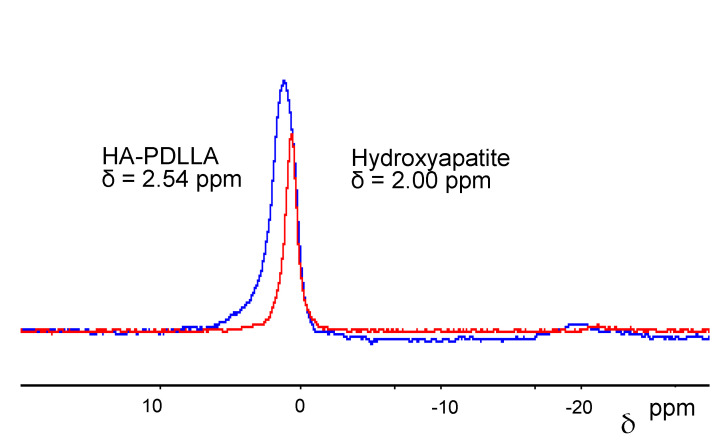
^31^P magic-angle spinning (MAS)-NMR of HA-PDLLA 10 (blue line) and HA (red line).

The attachment of the polymer to the surface of the hydroxyapatite particles could also be verified by ^31^P magic-angle spinning (MAS) NMR, 1D and 2D NMR. The ^31^P MAS NMR spectra for the surface-grafted hydroxyapatite and non-modified hydroxyapatite are shown in Figure 5. ^31^P NMR spectroscopy is particularly useful in revealing reaction pathways due to the pronounced sensitivity of the phosphorus atom to changes in its environment [31]. Due to the rather small hydroxyapatite content, a small monomer to catalyst ratio product, HA-PDLLA 10 ((M)/(C) = 10), was used in order to observe the signal. The recorded signal shows a downfield shift from 2.0 ppm in non-modified HA to 2.54 ppm in HA-PDLLA 10 indicating that the chemical environment of the phosphorus atom in the grafted HA crystal has changed. Moreover, the grafting link consists probably of Ca^2+^--^−^O-C(O)- association rather than a P-O-C(O)- bond, which is more labile and would cause a more dramatic chemical shift [32]. The observed downfield shift trend has been reported in previous publications [16], as well.

**Figure 6:**
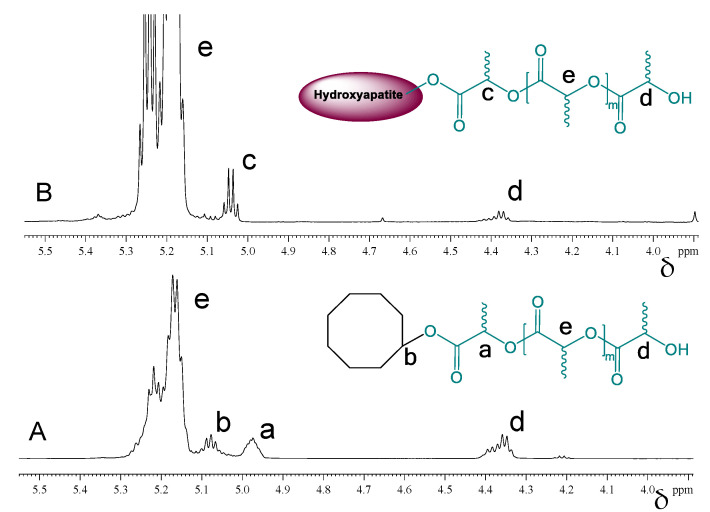
^1^H-NMR spectra in CDCl_3_of cyclooctanol-PDLLA (**A**) and HA-PDLLA (**B**).

The grafting of PDLLA onto the surface of HA crystals was further investigated by ^1^H- and ^13^C-NMR analyses. The overlaid ^1^H-NMR spectra shown in Figure 6 reveal the different chemical environment of the polymer’s methine group in close proximity to the initiator moiety. While the signals for the backbone- and end CH groups have the same chemical shifts (Figure 6, e and d), the neighboring cyclooctanol group and the surface Ca-O-C(O) groups in hydroxyapatite cause the signals at 4.98 ppm (Figure 6A, a) and 5.03 ppm (Figure 6B, c), respectively. In the case of cyclooctanol-PDLLA, however, there is one more characteristic methine group related to the cyclooctanol molecule, which appears at 5.09 ppm (Figure 6A, b).

**Figure 7:**
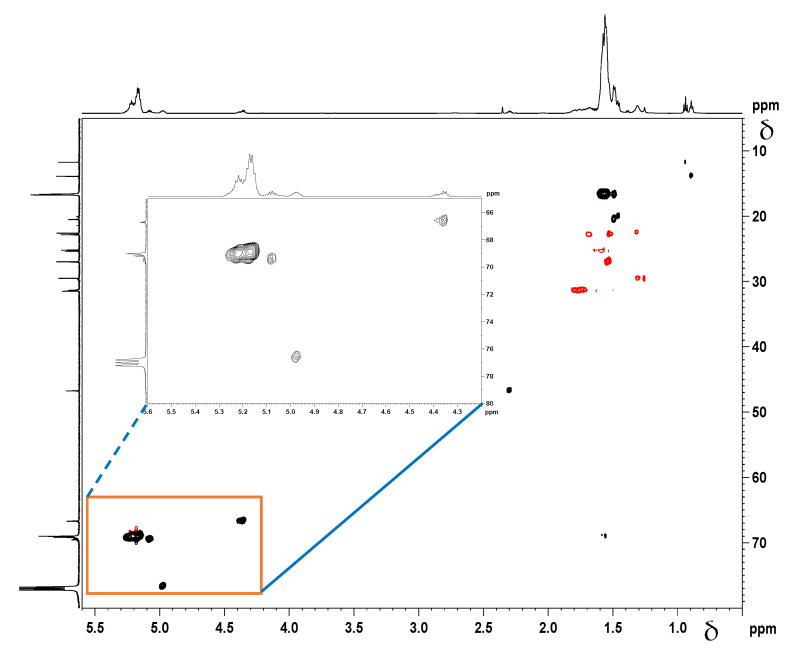
^1^H-^13^C Distortionless Enhancement of Polarization Transfer Heteronuclear Single Quantum Correlation (DEPT-HSQC) spectrum of the cyclooctanol-PDLLA homopolymer.

**Figure 8:**
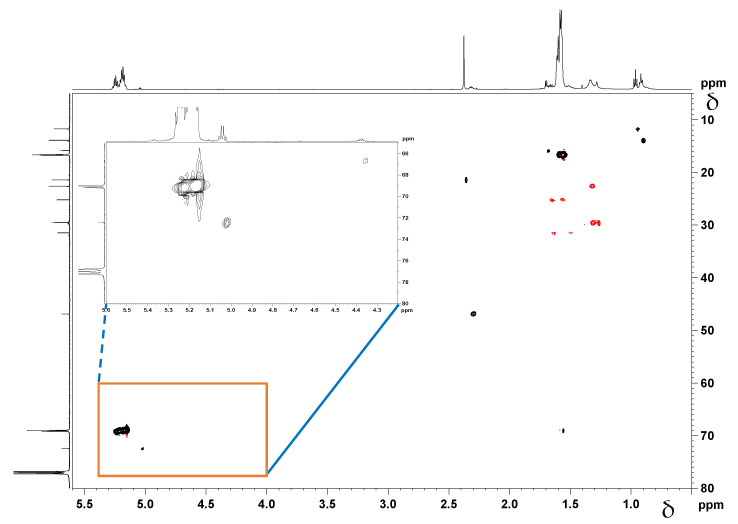
^1^H-^13^C DEPT-HSQC NMR spectrum of HA-PDLLA grafts.

Correlation NMR analyses (Figure 7 and Figure 8) were used to determine, which peaks originate from the –C(O)-***CH***(CH_3_)-O-groups next to the initiator moieties (cyclooctanol and hydroxyapatite, respectively). A Distortionless Enhancement of Polarization Transfer Heteronuclear Single Quantum Correlation (DEPT-HSQC) experiment was performed to determine the directly attached ^1^H-^13^C couplings. Four characteristic methine peaks at 4.35 ppm, 4.98 ppm, 5.09 ppm, and 5.15 ppm were detected for cyclooctanol-PDLLA compared to only three –CH peaks for hydroxyapatite-PDLLA at 4.35 ppm, 5.03 ppm, and 5.15 ppm, as shown in the insets of Figure 7 and Figure 8. The forth methine peak at 5.09 ppm in the cyclooctanol-PDLLA product is concluded to relate to the –(CH_2_)_2_-***CH***-O-C(O)- group in the initiator cyclooctanol (group b in the structure in Figure 6A).

**Figure 9:**
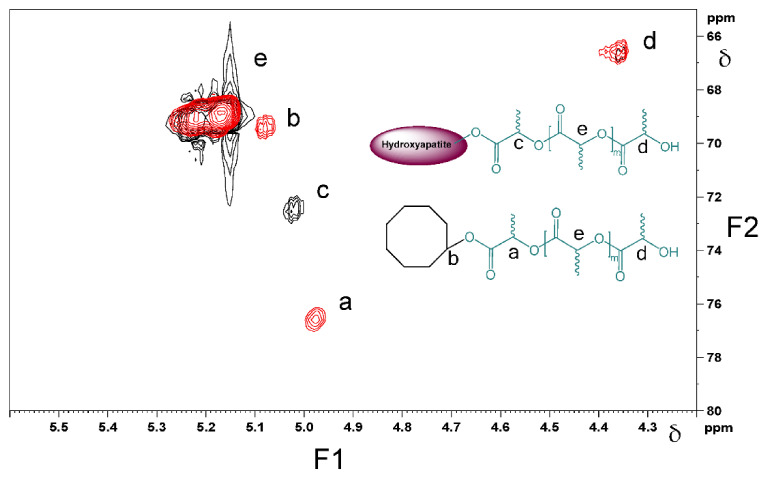
Overlay of the ^1^H-^13^C HMBC NMR spectra of cyclooctanol-PDLLA (red color) and HA-PDLLA (black color). F1 = conventional ^1^H spectrum; F2 = conventional ^13^C spectrum.

The assignment of the different methine peaks in both products was also supported by a long range ^1^H-^13^C heteronuclear correlation experiment, Heteronuclear Multiple Bond Coherence (HMBC), Figure 9. The methine peak emerging at δ = 4.35 ppm/ δ = 66.7 ppm (peak ‘d’ in Figure 9) is assigned to the –***CH***-OH group, which is the same end-group present in both cyclooctanol-PDLLA and HA-PDLLA. Another –CH- peak observed in both structures at δ = 5.15 ppm/ 69.5 ppm is concluded to be the –C(O)-***CH***(CH_3_)-O- group in the repeating units of the polymer chains (peak e in Figure 9). The methine groups in the polymer chain that are directly connected to initiator moieties, however, exhibit a different chemical environment, which is confirmed by the NMR analysis (peaks a and c in Figure 9). The overlay of the ^1^H-^13^C HMBC NMR spectra presented in Figure 9 clearly shows the ester linkage between the polymer and the initiator moieties.

**Figure 10:**
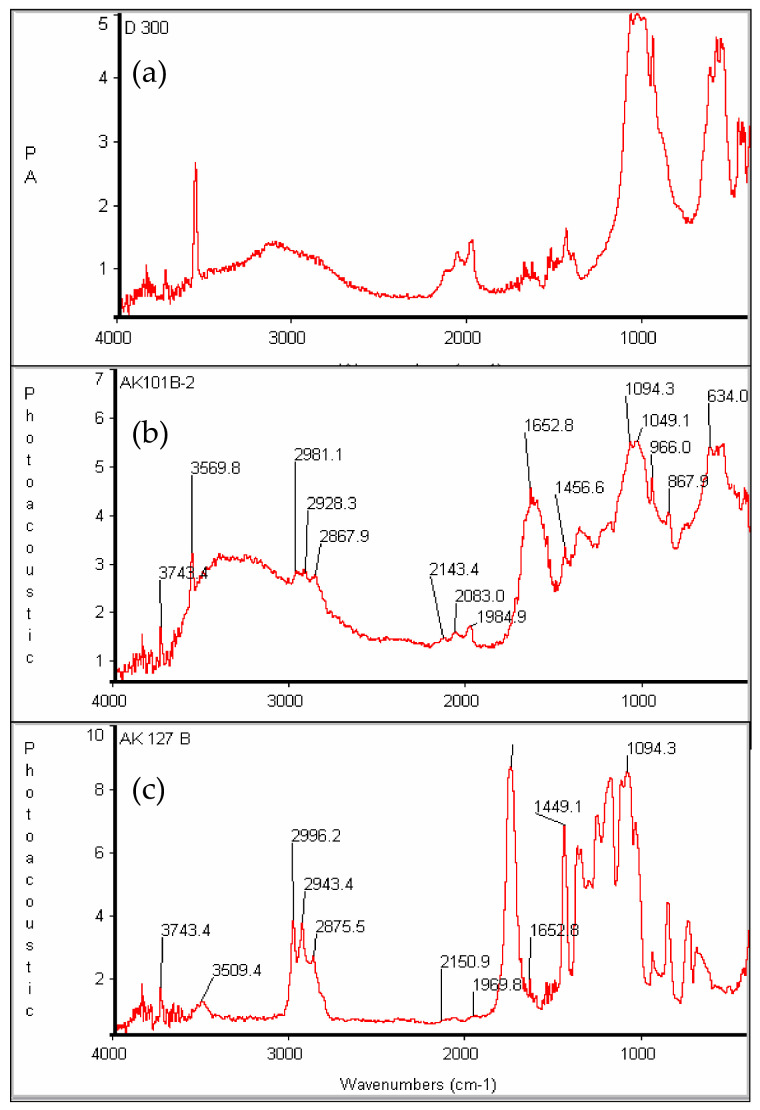
FT-IR spectra of (**a**) pure hydroxyapatite and HA-PDLLA formed by HA as the initiator with (**b**) (M)/(C) = 54 and (**c**) (M)/(C) = 217; see Table S1 for polymerization conditions.

The final evidence for the successful growth of PDLLA on the surface of the hydroxyapatite is provided by infra-red spectroscopy. Figure 10 contains the FT-IR spectra of hydroxyapatite and HA-PDLLA synthesized with different (M)/(C) ratios. The HA spectrum (Figure 10a) shows the typical absorption bands for PO_4_^3−^ at 600–650 cm^−1^ (antisymmetric bending), 960 cm^−1^ (symmetric stretching) and 1040–1094 cm^−1^ (antisymmetric stretching) [33] together with a typical combination of a sharp and a broad band between 3560 cm^−1^ and 3100 cm^−1^ due to hydrogen bonded HO^−^ [34]. Interestingly the position of the C=O band in the HA-PDLLA shifts from 1653 cm^−1^ for polymers produced at (M)/(C) = 19 and 54 (Figure S3 and Figure 10b) to a value more typical of the ungrafted PDLLA (1740 cm^−1^) for polymers from (M)/(C) = 217 (Figure 10c). At the same time the HA absorption bands gradually weaken. This unusual molecular mass dependence of the FT-IR absorbances of the ester groups could possibly be explained by their tethering at the surface of the HA particles due to some selective interaction with the negatively charged HA surface (the zeta potential of HA is estimated to be between −8 and −12 mV [35,36]. Moreover, at lower molecular mass (shorter chains) a significant portion of the carbonyl groups belong to the fraction of Ca^2+^---^−^O-C(O) linkages, characterized by an antisymmetric stretching vibration at 1653 cm^−1^ [36]. At monomer/initiator ratios of 100 and higher their relative content in the HA-graft would diminish and the spectrum would be dominated by the absorption bands, characteristic for the polyester carbonyl sufficiently removed from the HA surface to experience any selective interaction (and immobilization) with the negatively charged HA surface.

**Figure 11:**
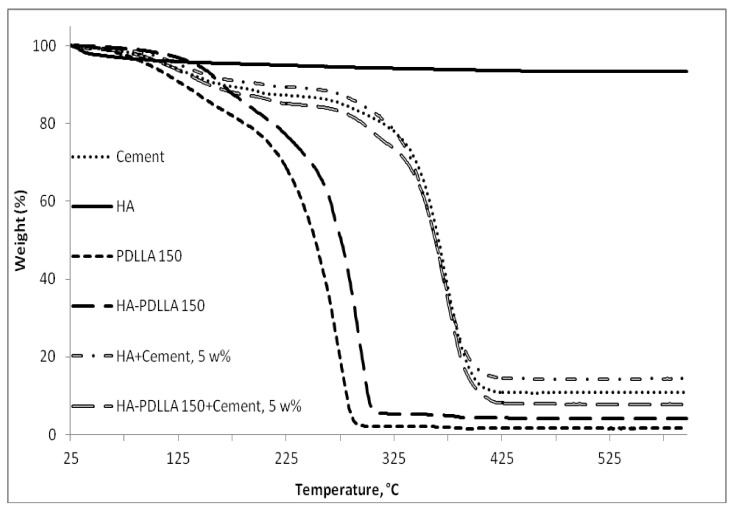
TGA thermograms of Simplex P SpeedSet radiopaque bone cement (Cement); hydroxyapatite (HA), poly(lactide) at (M):(C) = 150 (PDLLA 150), hydroxyapatite-polylactide grafts at (M):(C) = 150 (HA-PDLLA 150), and physical mixtures at 5% weight ratio of: hydroxyapatite + Simplex P SpeedSet (HA + Cement) and HA-PDLLA 150 + Simplex P SpeedSet (HA-PDLLA 150 + Cement).

**Figure 12:**
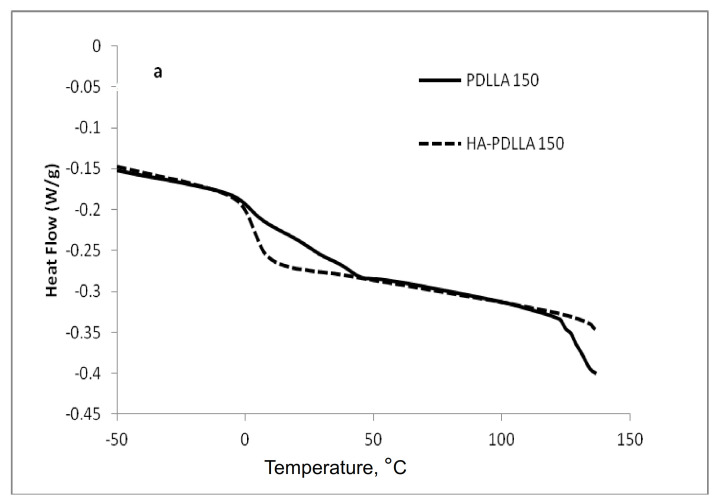

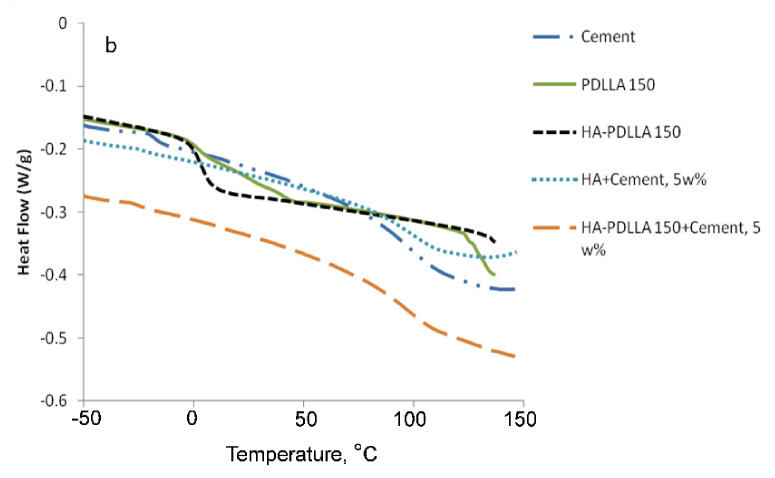
DSC traces of (**a**) poly(lactide) at (M):(C) = 150 (PDLLA 150), hydroxyapatite-polylactide grafts at (M):(C) = 150 (HA-PDLLA 150); (**b**) radiopaque bone cement Simplex P SpeedSet (Cement) and physical mixtures of: hydroxyapatite (5% weight) + Simplex P SpeedSet (HA + Cement) and HA-PDLLA 150 (5% weight) + Simplex P SpeedSet (HA-PDLLA 150 + Cement).

HA-PDLLA degradation behavior and phase compatibility in mixtures with commercially available PMMA based bone cement (Simplex P Speedset, Stryker Orthopaedics) were investigated through TGA and DSC, respectively. An example with physical mixtures of 5% weight HA-PDLLA 150 in bone cement is shown in Figure 11 and Figure 12a,b. The simple physical mixture of the grafts with Simplex P Speedset does not deteriorate the thermal stability of the cement. Furthermore, the observed DSC curve for the same mixture exhibits the glass transition behavior of the original bone cement formulation (Figure 12b). Hence, the modification of standard bone cement formulations with 5% weight HA-PDLLA grafts will not compromise the cement stability and storage requirements.

**Figure 13:**
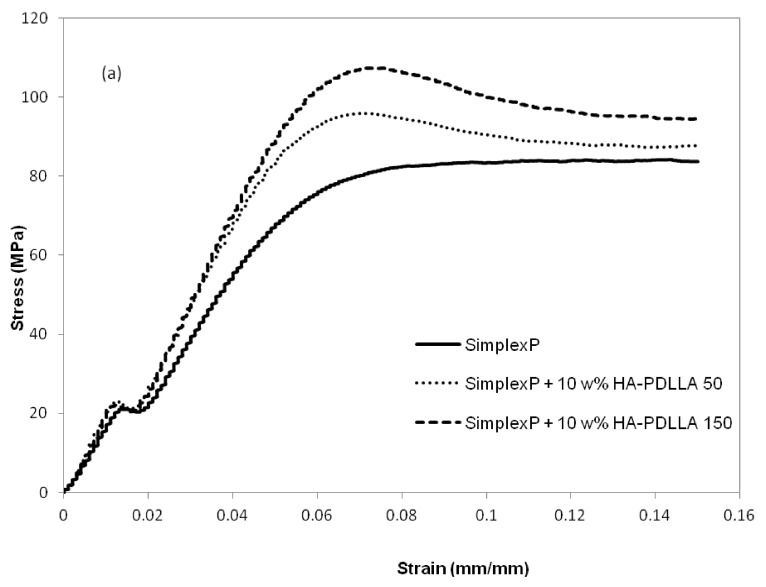

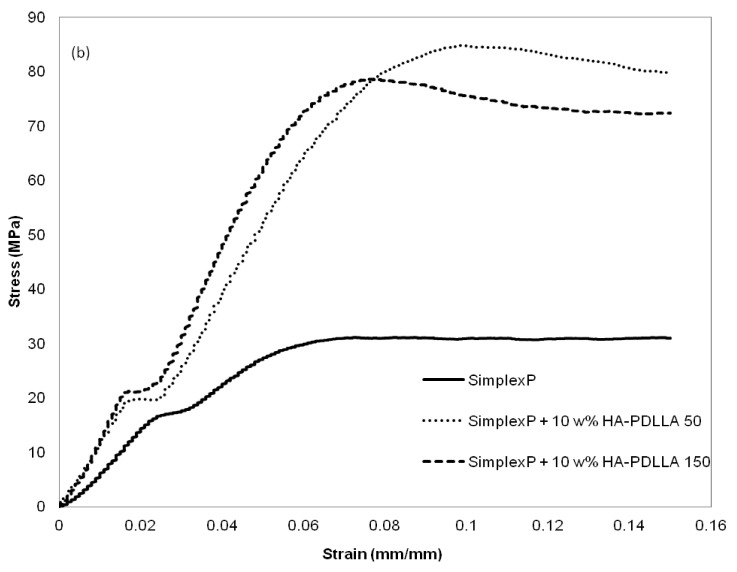
Compression stress-strain curves for standard and modified bone cement formulations exposed to aqueous medium at pH 4. (**a**) control, 0 weeks; (**b**) after exposure for 4 weeks. See Figure S4 for the compression stress–strain curves of the same formulations after exposure for 8 weeks.

The mechanical properties at different environmental conditions were investigated with two HA-PDLLA nanografts (Table 1, (M)/(C) = 50 and 150). The compression stress–strain curves of the cement compositions before and after 4 weeks of exposure to an aqueous medium at pH 4 are shown in Figure 13a,b.

The linear elastic portion of each curve was used for the experimental determination of the compression modulus for the standard and the modified bone cements. The compressive strength was calculated as the failure load (the load at the 2.0% offset) divided by the corresponding cross-sectional area.

**Figure 14:**
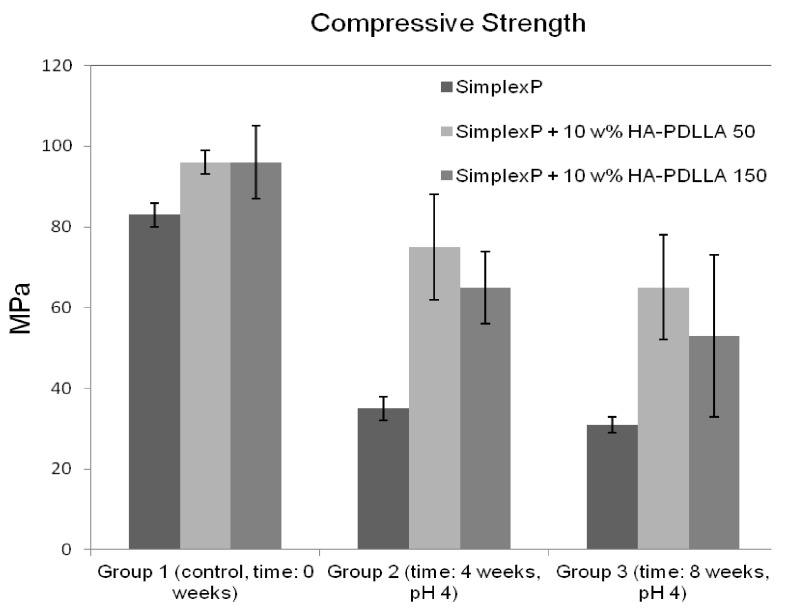
Mean (±SD) compressive strength of standard (Simplex P) and modified bone cement formulations tested before (control) and after accelerated degradation in an acidic solution (pH = 4) for 4 and 8 weeks.

The compressive strengths of all three cement formulations in the control group exceeded the minimum of 70 MPa as required by ASTM F451-99a (see Materials and Methods for description), Figure 14. The compressive strengths for the modified bone cement specimens were observed to be higher than those for the standard cement formulation. After 4 weeks of accelerated degradation in an acidic solution, the observed compressive strengths were 35 ± 3 MPa for the plain bone cement, 75 ± 13 MPa for the cement with 10% weight HA-PDLLA 50, and 65 ± 9 MPa for the formulation with 10% weight HA-PDLLA 150. The exhibited loss of compressive strength was in the amount of 58% for the SimplexP bone cement specimens, 22% for SimplexP + 10% weight HA-PDLLA 50, and 32% for SimplexP + 10% weight HA-PDLLA 150. After 8 weeks of accelerated degradation in an acidic solution, the observed compressive strengths were 31 ± 2 MPa for the plain bone cement, 65 ± 13 MPa for the cement with 10% weight HA-PDLLA 50, and 53 ± 20 MPa for the formulation with 10% weight HA-PDLLA 150. The compressive strength loss was calculated to be 63% for the SimplexP standard formulation, 32% for the SimplexP + 10% weight HA-PDLLA 50 formulation, and 45% for the SimplexP + 10% weight HA-PDLLA 150 formulation. The mean compressive strengths for the three cement formulations were all significantly different from one another (*p* < 0.05).

**Figure 15:**
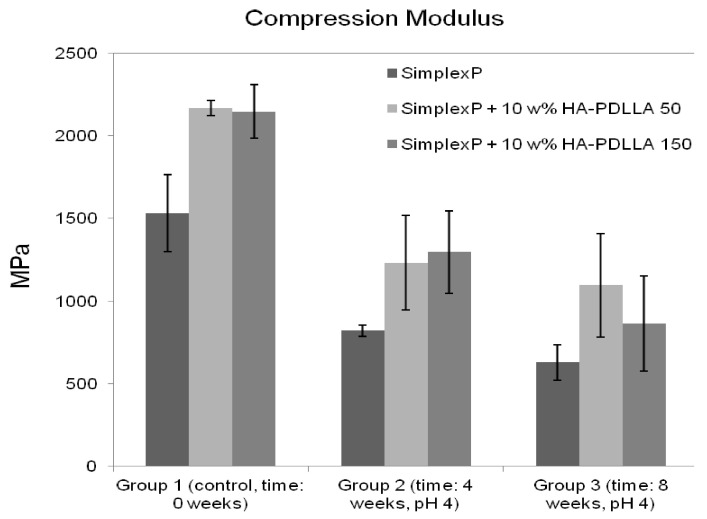
Mean (±SD) compression modulus of standard (Simplex P) and modified bone cements tested before (control) and after immersion in an acidic solution (pH = 4) for 4 and 8 weeks.

The compression modulus for the standard and modified bone cements are presented in Figure 15. All three cement formulations exhibited a marked decrease in stiffness after 4 weeks of immersion in an acidic solution. The compression modulus was observed to decrease by 46% for the standard bone cement specimens, 43% for the specimens containing 10% weight HA-PDLLA 50, and 40% for the specimens with 10% weight HA-PDLLA 150. After 8 weeks of accelerated degradation, however, the loss of stiffness for the test specimens modified with 10% weight HA-PDLLA 50 was observed to be lower compared to that of either the SimplexP or the SimplexP + 10% weight HA-PDLLA 150 formulation (49% vs. 59% and 60%, respectively). The mean values of the compression modulus for all three cement formulations were significantly different from one another (*p* < 0.05).

The results of this study indicate that the compression properties of PMMA-based bone cement are improved by the addition of HA-PDLLA polymers. Both the compressive strength and the compression modulus of SimplexP test specimens measured in our experiments were in accordance with previously published values [2]. According to the referenced review, the compressive strength of SimplexP specimens, prepared manually and tested 24 h after preparation, is reported to be 89.2 MPa; the reported compression modulus is 2540 MPa. We found that adding HA-PDLLA 50 or HA-PDLLA 150 polymeric grafts to SimplexP resulted in an increase in the observed compressive strength and stiffness of the bone cement specimens. The dissolution of the additives in the monomer liquid prior to the preparation of the cement specimens very likely resulted in their homogeneous distribution in the polymer matrix and prevented the formation of agglomerates. Another factor affecting the regular distribution of the additives in the polymer matrix is the chemical linkage between HA and PDLLA. The PDLLA chains attached to the HA surface are fully compatible with the organic polymer matrix; the organic nature of both the additives and the acrylic cement allowed for a regular mixing of these components.

The compression properties of the standard bone cement and those of the modified cements decreased significantly after being subjected to degradation in extreme conditions (i.e., at pH 4). Interestingly, the observed loss of compression strength for the HA-PDLLA 50 modified bone cements after 8 weeks of accelerated degradation was one half of that measured for the plain bone cement (Figure 14). One possible explanation for this observation is the sample porosity. The presence of pores is an important factor that significantly affects the compression properties of bone cements. Typically, low porosity materials have stress–strain curves with steep initial slopes of the linear portion (below 2% strain). The stress–strain curves of high porosity materials, by contrast, exhibit more shallow initial slopes. Our results showed similar characteristic features of the stress–strain curves for the modified and the plain bone cements subjected to accelerated degradation (Figure 13b and Figure S4c). The pore formation was strongly related to the degradation processes occurring in the polymer matrix. Under acidic conditions, both PMMA and PDLLA would undergo hydrolysis of the ester bonds; however, in the case of PDLLA, the ester linkages subject to hydrolysis were part of the polymer backbone. In the case of PMMA, the product of the acidic hydrolysis, methanol, was miscible in water and would possibly leak out easily from the cement specimens. Another by-product of the PMMA hydrolysis, methacrylic acid, was also miscible in water. HA-PDLLA, on the other hand, was degraded by random chain scission and the initially resulting oligomers with a high molecular weight were not miscible in water.

**Figure 16:**
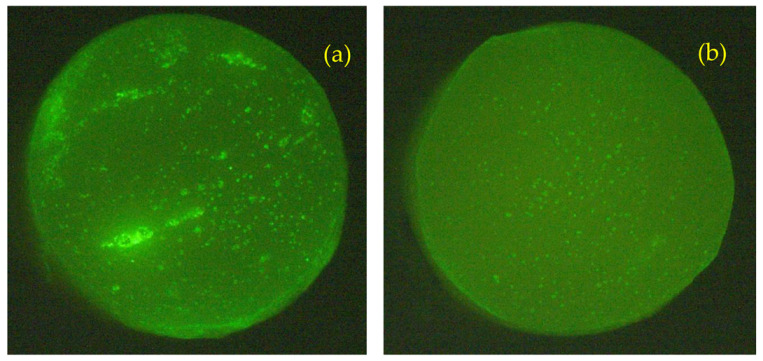
Cross sections of standard bone cement ((**a**), Simplex P) and a modified formulation ((**b**), Simplex P + HA-PDLLA 50) photographed under UV light after accelerated degradation in an acidic solution (pH = 4) for 4 weeks. Diameter of specimens: 6 mm. Please note the even higher pore distribution in the modified bone cement specimen (**b**).

Our hypothesis is that the leakage of the hydrolysis byproducts would cause the formation of void spaces (micropores) in the cement matrix and would result in the loss of both strength and stiffness of the material. The modified bone cement, however, exhibited a lower decrease in the measured compression properties. One possible explanation for this observation was the presumed tendency of the initially formed oligomers with higher molecular mass to not mix with the water medium and to remain in the cement matrix due to hydrophobic–hydrophobic interactions. Moreover, when the water diffused in the cement bulk, the volume of the PDLLA portion of the polymer additives would notably increase in accordance with the water absorption behavior of PDLLA, as previously reported [37]. Accordingly, the expanded volume of the polymer grafts would result in “filling” some of the pores in the cement. Representative pictures of plain cement specimens and specimens with 10% weight HA-PDLLA 50 after incubation at pH 4 for 4 weeks are shown in Figure 16. The difference in number and size of pores in the specimen cross sections is clearly seen. Similar results were observed also for Simplex P + 10% weight HA-PDLLA 150 under identical conditions (Figure S6).

That is why the compressive strength and stiffness of the modified bone cement specimens were higher compared to those of the standard formulation under the described experimental conditions.

This preliminary investigation of HA-PDLLA modified acrylic bone cement was limited to the mechanical properties of bone cement in compression. The compressive strength measurement is a critical step for determining whether the modified formulations will be strong enough for clinical applications. However, other mechanical tests (i.e., tensile strength and fracture toughness) have to be performed for a more complete and detailed characterization of HA-PDLLA—containing bone cements.

## 3. Materials and Methods

**Materials.** Toluene (‘Baker analyzed^®^ HPLC solvent, 0.01% H_2_O) was purchased from Mallinckrodt Baker, Inc. (Phillipsburg, NJ, USA) and freshly distilled over CaH_2_ under argon before use. Hydroxyapatite, HA, (synthetic powder, reagent grade), cyclooctanol (94%), and stannous 2-ethylhexanoate (SnOct_2_) were purchased from Sigma-Aldrich, Inc. (St. Louis, MO, USA); d,l-lactide (min. 99.5%) was purchased from Polysciences, Inc. All reagents were stored in a dry glovebox system operating on inert gas (Ar) upon opening and used without further purification. Tetrahydrofuran (THF, HPLC grade, stabilized with BHT, 0.05% H_2_O) was obtained from Pharmco-Aaper. Chloroform-*d* (D, 99.8%) was purchased from Cambridge Isotope Laboratories, Inc. (Andover, MA, USA) and stored refrigerated on molecular sieves. Radio-opaque bone cement “Simplex P Speedset” from Stryker Orthopaedics (Mahwah, NJ, USA) was stored and processed according to company’s instructions.

**Polymerization procedure.** All reaction mixtures were prepared inside a dry glovebox SYS1-2GB (Innovative Technology, Inc., Newburyport, MA, USA). All polymerizations were conducted in dry toluene for 3 h at reflux (~111−112 °C). Two series of ring-opening polymerizations of d,l-lactide were conducted: (a) d,l-lactide + SnOct_2_ + cyclooctanol with variation of the monomer to catalyst (M/C) ratio; (b) d,l-lactide + SnOct_2_ + hydroxyapatite with variation of the M/C ratio.

A typical polymerization procedure was performed as follows: d,l-lactide (0.5693 g, 3.95 mmol) and hydroxyapatite (0.03 g, 5.97 × 10^−2^ mmol) were weighed into a double neck round bottom flask (50 mL), equipped with a magnetic stirring bar. The flask was attached to a condenser with a Dean-Stark trap and an inlet adaptor for inert gas, and the side neck was closed with a rubber septum. A flow of dry argon was continuously passed through the reaction mixture. The solvent (toluene, 10 weight %) and the catalyst (SnOct_2_, 7.9 × 10^−2^ mmol) were transferred to the flask by a gas tight syringe. The temperature of the oil bath was increased to 111 °C and kept in the range of 111–112 °C for 3 h. The resulting reaction mixture was a viscous liquid. The material was precipitated in cold methanol and the residual solvent was evaporated using a rotary evaporator. The obtained product was a white-colored foamy material. Yield: 0.6034 g (94%). The molecular mass characteristics of the material were determined by size exclusion chromatography. M_n_ = 3800 Da, Ð = 2.51 (Table 1).

**Cement Preparation.** Three different cement compositions were prepared using Simplex™ P SpeedSet™ cement (Mahwah, NJ, USA) and HA-PDLLA additives: (a) Simplex™ P SpeedSet™ bone cement; (b) Simplex™ P SpeedSet™ with 10% *v*/*v* HA-PDLLA 50; (c) Simplex™ P SpeedSet™ with 10% *w*/*w* HA-PDLLA 150. The cement specimens were prepared according to the instructions provided by the manufacturer. The polymer additives in the amount of 10% *w*/*w* were first dissolved in the liquid monomer at room temperature. Then, the polymer powder and the monomer liquid were hand-mixed in an inert container and stirred with a spatula for 2 min. When a dough-like mass was formed, the mixture was manipulated by hand for 2 more minutes and packed into a cylindrical-shaped mold.

**Preparation of Samples for Mechanical Testing.** The casting of compression samples was performed according to ASTM standard F451-99a [38]. The three cement compositions were prepared and put into a Teflon mold consisting of cylindrical holes (each 6 mm in diameter and 12 mm in height). The specimens were allowed to polymerize in the mold for 1 h, followed by 24 h of curing out of the mold. All specimens were polished with 280, 320, and 600 grit sand papers. The specimens were visually examined for surface defects and only acceptable specimens with a uniform appearance and no visible surface defects were used in the study (See Figure S4 for an example).

**Degradation Experiments.** The effect of the physiological medium on the properties of the standard and modified bone cements was investigated by exposing the specimens to a solution with fixed acidic pH (pH = 4, in house preparation). The test specimens were grouped according to the duration of accelerated degradation as follows: Group 1, control, degradation time 0 weeks; Group 2, degradation time 4 weeks; Group 3, degradation time 8 weeks. Six specimens of each group were immersed in a 5 mL acidic solution and incubated in an incubator shaker at 37 °C for 4 and 8 weeks, respectively. At the end of each degradation period the specimens were dried to a constant weight and subjected to a compression test.

**Methods.** Size exclusion chromatography (SEC) was performed on a system (Waters Corporation, Milford, MA, USA) consisting of a Waters M510 HPLC pump, Waters U6K injector and Viscotek 250 detector (Viscotek, Malvern Panalytical Inc., Westborough, MA, USA). The separation was achieved at 40 °C on a set of three 5 µm PLgel columns (Polymer Laboratories, Inc., Amherst, MA, USA) with pore sizes of 50 Ǻ, 1000 Ǻ, and mixed C and eluent flow rate of 0.8 mL/min. Typically, 5 mg of material were dissolved in 1 mL freshly distilled THF and then filtered through a 0.45 µm PTFE filter before injection. The columns were calibrated with a set of 41 poly(styrene), PSt, standards (Polymer Standards Service-USA, Inc., Warwick, RI) with narrow dispersity and molecular masses between 162 and 1,670,000 Da. All chromatograms were acquired by the OmniSEC 3.1 software package (Viscotek, Malvern Panalytical Inc., Westborough, MA, USA) and the molecular masses were calculated using conventional PSt calibration. The chromatograms shown in Figure 1 and Figure S1 were produced using a set containing Waters Styragel linear 4E and 5E columns in series under identical analysis and calculation protocols.

Thermogravimetric analysis, TGA, was performed on a Hi-Res TGA 2950 thermogravimetric analyzer (TA Instruments, Waters Corporation, Milford, MA, USA) under a nitrogen atmosphere between 25 °C and 600 °C at a heating gradient of 20 °C/min. The degradation temperature was calculated at the onset of the weight loss.

Differential scanning calorimetry, DSC, was conducted using a differential scanning calorimeter DSC Q200 V24.4 (TA instruments, Waters Corporation, Milford, MA, USA), equipped with a refrigerated cooling system. Samples (5–8 mg) were crimped in Tzero aluminum pans and scanned at 10 °C/min in the range 25–140 °C under a continuously purged dry nitrogen atmosphere (flow rate of 50 mL/min). A heating–cooling–heating cycle was used. The data were collected and analyzed using TA Instruments Universal Analysis 2000 V4.3A software (TA Instruments, Waters Corporation, Milford, MA, USA). The glass transition temperatures (T_g_) were calculated at the inflection point of the two baseline tangents before and after the transition using the second heating stage.

FT-IR analyses were performed on Nicolet Model 400 spectrophotometer (Nicolet, ThermoFisher Scientific, Waltham, MA, USA) for soluble samples by depositing a drop of polymer solution in dry CHCl_3_ on a ZnSe Infracal plate (Wilks, Spectro Inc., Littleton, MA, USA).

Nuclear magnetic resonance (NMR) spectra were obtained using a Bruker AVANCE 600 MHz spectrometer (Bruker, Inc., Billerica MA, USA) operating at 25 °C, with chloroform-*d*_1_ (CDCl_3_) as the solvent and internal standard. The ^31^P-NMR spectra were obtained in solid state using 4 mm DSI CPMAS probe rotors (DOTY Scientific, Columbia, SC, USA) under proton decoupling of 35 kHz, a recycle delay of 2s, and magic-angle spinning (MAS) at a frequency of 6.2 kHz. The DEPT edited HSQC analyses used previously described protocol [39]: delay time 2 s, obtaining 256 increments with 16 scans per increment, within spectral widths of 7212 Hz (^1^H) and 25 000 Hz (^13^C) and operating at 125 and 600 MHz. The HMBC analyses were executed using a delay time of 1.2 s, obtaining 256 increments with 40 scans per increment, within spectral widths of 4807 Hz (^1^H) and 36,000 Hz (^13^C) operating at 125 and 600 MHz.

The compression test was carried out using a screw-driven mechanical load frame with a 500 kg capacity load cell (Q-test, MTS Systems, Eden Prarie, MN, USA). Six specimens (Figure S4) of each composition (standard and modified at 10% *w*/*w* additive bone cements) were tested in air at room temperature at a displacement rate of the cross head of 25.4 mm/min. The compressive strength and modulus were calculated using TestWorks 4.0 software (MTS Systems Co.7, Eden Prairie, MN, USA).

## 4. Conclusions

The experimental results of the present work demonstrate that the surface hydroxyl groups of hydroxyapatite particles can be used as initiator in the ring-opening polymerization of d,l–lactide resulting in efficient and reproducible growth of poly(d,l-lactide) chains onto the surface of HA. The covalent chemical bonding between HA and PDLLA was confirmed by SEC, TGA, DSC, FT-IR and NMR analyses. The addition of HA-PDLLA 50 and HA-PDLLA 150 improved the mechanical properties of commercial bone cement Simplex™ P SpeedSet™, more noticeably after accelerated degradation. The prepared grafts are a novel formulation that combines the osteoconductivity and biocompatibility of HA with the biodegradability and resorbability of PDLLA. This combination could make these materials rather promising additives to existing bone cement formulations, which possess the ability for gradual and controlled degradation, resulting in a slowly progressing network of pores and inducing new bone growth. The occurrence of these processes and their effect on the mechanical properties of the bone cement are currently under investigation in our group.

## Figures and Tables

**Table 1:** Polymerization of d,l-lactide in toluene at 111 °C for 3 h with Cyclooctanol/Sn(Oct)_2_ or Hydroxyapatite/Sn(Oct)_2_ and different (Monomer)/(Catalyst) ratios.

(M)/(C)	PDLLA ^1^ Yield (%)	PDLLA M_n_ (Da) ^2^	Ð ^3^	HA-PDLLA ^4^ Yield (%)	HA-PDLLA M_n_ (Da) ^2^	Ð ^3^
10	93	540	1.19	95	1200	2.51
50	93	1200	1.61	94	3800	2.51
100	95	2500	2.09	96	5500	3.47
150	94	3100	2.54	97	5700	4.10
200	97	4250	3.24	97	6100	2.14

^1^ Cyclooctanol/Sn(Oct)_2_; ^2^ molecular mass determined by size exclusion chromatography (SEC) against PSt standards; ^3^ Ð-dispersity index (M_w_/M_n_); ^4^ hydroxyapatite/Sn(Oct)_2_.

## Data Availability

The data obtained by this study are included in this article and in the Supplementary Materials.

## Supplementary Materials

The following are available online, Table S1: Polymerization of D,L-lactide in triethylene glycol dimethyl ether (triglyme) at 130 °C for 48 h with Hydroxyapatite/Sn(Oct)_2_ and different (Monomer)/(Catalyst) ratios; Figure S1: SEC eluograms of the polymerization mixtures obtained in toluene at 111 °C after 3 h with D,L-lactide and HA/Sn(Oct)_2_ (green traces, 1) or Cyclooctanol/Sn(Oct)_2_ (red traces, 2) with (M)/(C) = 50 (a) and (M)/(C) = 100 (b); Figure S2: DSC thermograms of polyesters obtained with cyclooctanol (PDLLA 50 and PDLLA 200) and with hydroxyapatite (HA-PDLLA 50 and HA-PDLLA 200; Figure S3: FT-IR spectra of HA-PDLLA formed by HA/Sn(Oct)_2_ with (M)/(C) = 19 (a) and 540 (b); see Table S1 for polymerization conditions; Figure S4: Specimen for compression stress–strain testing before sand paper polishing; Figure S5: Compression stress–strain curves for standard and modified bone cement formulations exposed to aqueous medium at pH 4. (c) After exposure for 8 weeks; Figure S6: Cross sections of standard bone cement (a, Simplex P) and a modified formulation (b, Simplex P + HA-PDLLA 50. Photographs made after accelerated degradation in an acidic solution (pH = 4) for 4 weeks.

## Sample Availability

Samples of the compounds are not available from the authors.
