# Supplementary material for: Hydroxyapatite-poly(d,l-lactide) Nanografts. Synthesis and Characterization as Bone Cement Additives
Source: Molecules. 2021 Jan 15;26(2):424. doi: 10.3390/molecules26020424 (PMC7830310; doi:10.3390/molecules26020424)
Supplement: Supplementary file 1 [file molecules-26-00424-s001.pdf]

Supplementary Materials

Hydroxyapatite-Poly(D,L-lactide) Nanografts. Synthesis and Characterization as Bone Cement Additives

Kristina L. Goranova [1], Anne Kathrine Kattenhøj Sloth Overgaard [1,2] and Ivan Gitsov [1,3,*]

[1] Department of Chemistry, State University of New York-ESF, Syracuse, NY 13210, USA; klgorano@syr.edu (K.L.G.); akovergaard@yahoo.dk (A.K.K.S.O.)
[2] Coloplast, Holtedam 1-3, DK-3050 Humlebǽk, Denmark
[3] The Michael M. Szwarc Polymer Research Institute, Syracuse, NY 13210, USA
* Correspondence: igivanov@syr.edu; Tel.: +1-315-470-6860
Received: 22 December 2020; Accepted: 6 January 2021; Published: 15 January 2021

Contents

Table S1. Polymerization of D,L-lactide in triethylene glycol dimethyl ether (triglyme) at 130°C for 48 h with Hydroxyapatite/Sn(Oct)$_2$ and different [**M**onomer]/[**C**atalyst] ratios…………………………S2

Figure S1. SEC eluograms of the polymerization mixtures obtained in toluene at 111°C after 3 h with D,L-lactide and HA/Sn(Oct)$_2$ (green traces, 1) or Cyclooctanol/Sn(Oct)$_2$ (red traces, 2) with [M]/[C] = 50 (a) and [M]/[C] = 100 (b)..S3

Figure S2. DSC thermograms of polyesters obtained with cyclooctanol (PDLLA 50 and PDLLA 200) and with hydroxyapatite (HA-PDLLA 50 and HA-PDLLA 200)……………………………………………………S4

Figure S3. FT-IR spectra of HA-PDLLA formed by HA/Sn(Oct)$_2$ with [M]/[C] = 19 (a) and 540 (b); see Table S1 for polymerization conditions……………………………………………………………………………………S5

Figure S4. Specimen for compression stress-strain testing before sand paper polishing

Figure S5. Compression stress-strain curves for standard and modified bone cement formulations exposed to aqueous medium at pH 4. (c) after exposure for 8 weeks……………………………………………………S6

Figure S6. Cross sections of standard bone cement (a, Simplex P) and a modified formulation (b, Simplex P + HA-PDLLA 50. Photographs made after accelerated degradation in an acidic solution (pH = 4) for 4 weeks………………S7

Table S1. Polymerization of D,L-lactide in triethylene glycol dimethyl ether (triglyme) at 130°C for 48 h with Hydroxyapatite/Sn(Oct)$_2$ and different [**M**onomer]/[**I**nitiator] ratios.

[M]/[C]	Sample Name	HA-PDLLA Yield	HA-PDLLA M_n (Da)[1]	HA-PDLLA M_w (Da)[1]
19	AK95A	11.9 %	n/a	n/a
19	AK101A	12.9 %	n/a	n/a
20	AK71A	22.7 %	n/a	n/a
20	AK77A	22.8 %	n/a	n/a
54	AK95B	11.4 %	n/a	n/a
54	AK101B	12.2 %	n/a	n/a
108	AK137A	12.3 %	n/a	n/a
108	AK171A	41.2 %	n/a	n/a
112	AK147A	18.0 %	n/a	n/a
162	AK137B	21.7 %	n/a	n/a
217	AK127B	19.2 %	n/a	n/a
540	AK127A	38.2 %	n/a	n/a

[1] (n/a) - not analyzed due to low yield

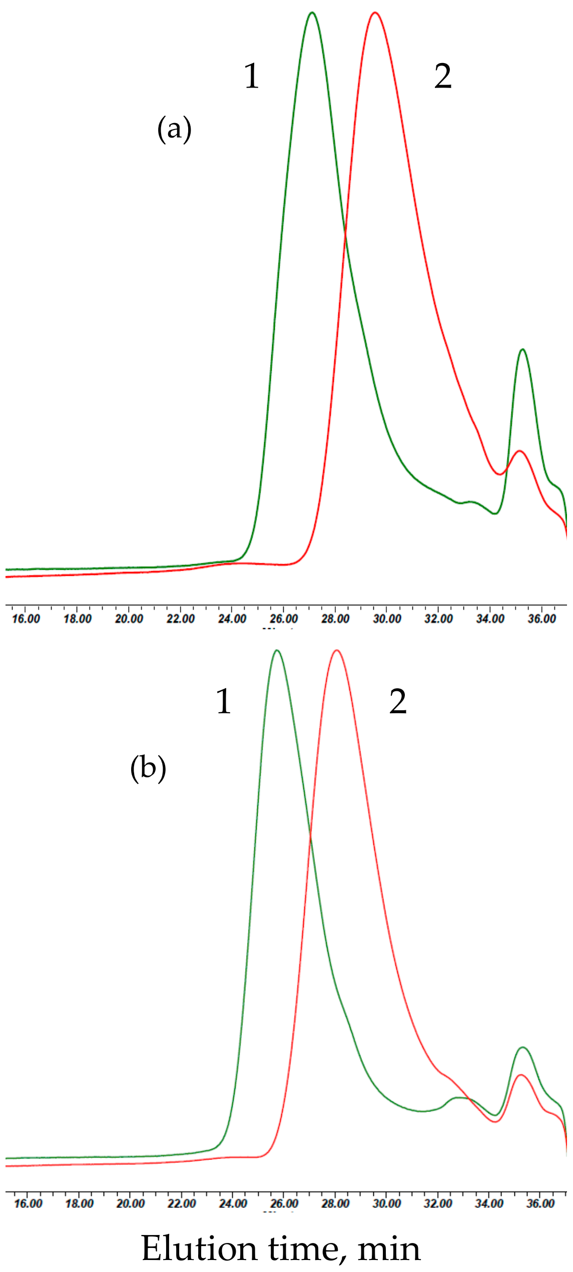

Figure S1. SEC eluograms of the polymerization mixtures obtained in toluene at 111°C after 3 h with D,L-lactide and HA/Sn(Oct)$_2$ (green traces, 1) and Cyclooctanol/Sn(Oct)$_2$ (red traces, 2) with (a) [M]/[C] = 50 and (b) [M]/[C] = 100.

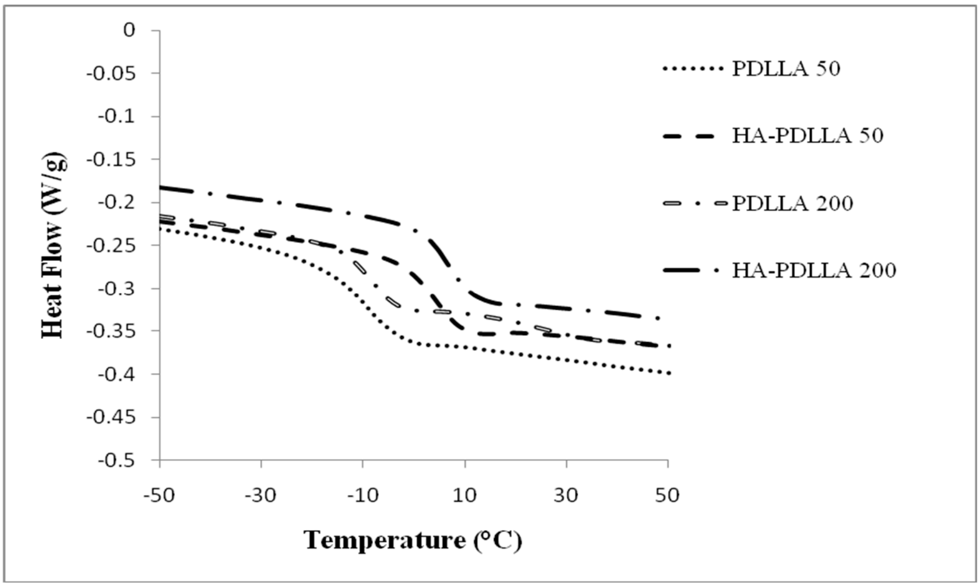

Figure S2. DSC thermograms of polyesters obtained with cyclooctanol (PDLLA 50 and PDLLA 200) and with hydroxyapatite (HA-PDLLA 50 and HA-PDLLA 200).

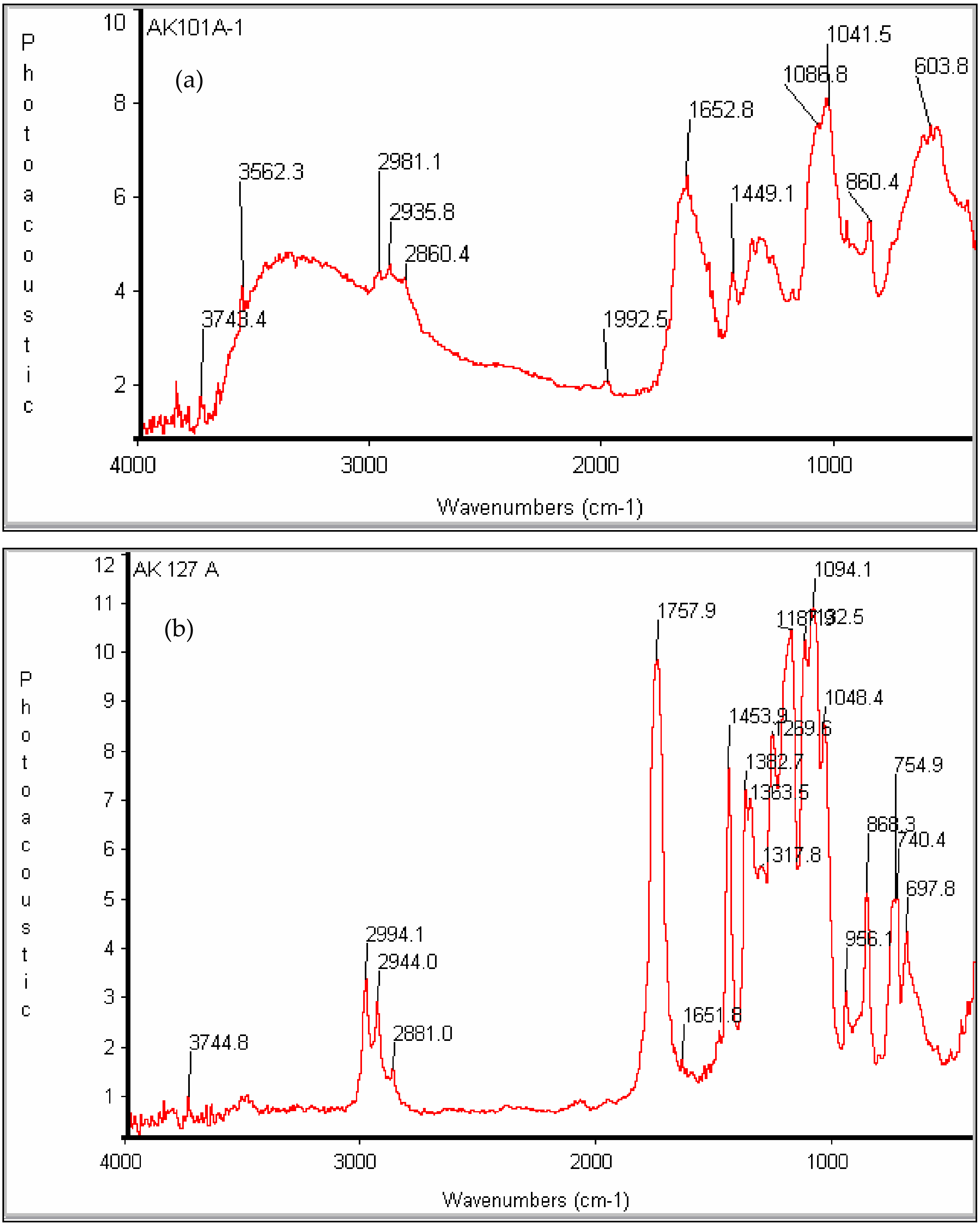

Figure S3. FT-IR spectra of HA-PDLLA formed by HA/Sn(Oct)$_2$ with [M]/[C] = 19 (a) and 540 (b); see Table S1 for polymerization conditions.

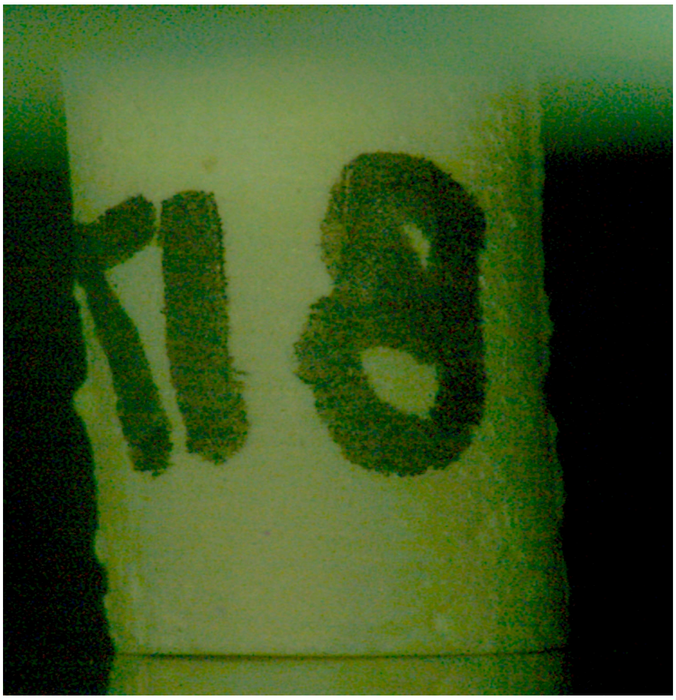

Figure S4. Specimen for compression stress-strain testing before sand paper polishing

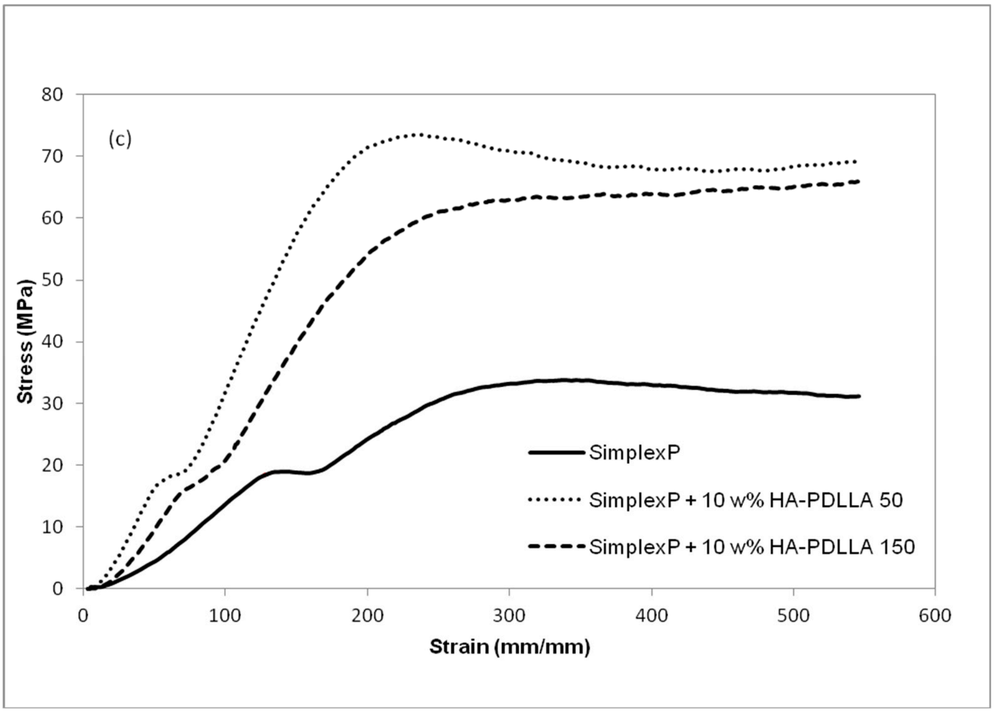

Figure S5. Compression stress-strain curves for standard and modified bone cement formulations exposed to aqueous medium at pH 4. (c) After exposure for 8 weeks.

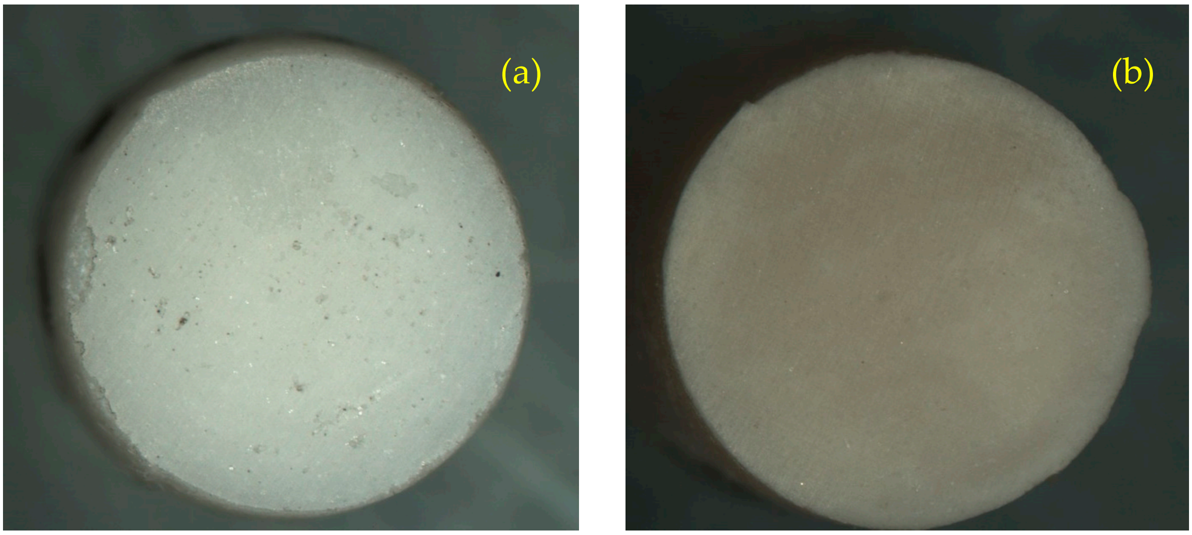

Figure S6. Cross sections of standard bone cement (a, Simplex P) and a modified formulation (b, Simplex P + HA-PDLLA 150. Photographs made after accelerated degradation in an acidic solution (pH = 4) for 4 weeks
